# Dietary Inflammatory Index and All-Cause Mortality in Older Adults with Hypertension: Results from NHANES

**DOI:** 10.3390/jcm12020506

**Published:** 2023-01-07

**Authors:** Yang Cao, Pengxiao Li, Yan Zhang, Miaohan Qiu, Jing Li, Sicong Ma, Yudong Yan, Yi Li, Yaling Han

**Affiliations:** 1 The Department of Cardiology, General Hospital of Northern Theater Command, Shenyang 110000, China; 2 The Department of Cardiology, Xijing Hospital, Air Force Medical University, Xi’an 710000, China

**Keywords:** dietary inflammatory index, hypertension, old adults, prognosis, NHANES

## Abstract

Both diet and inflammation are strongly associated with hypertension. However, the relationship between the dietary inflammatory index (DII) and the prognosis of hypertensive patients over 65 years of age is unclear. The objective of this study is to investigate the correlation between DII and all-cause mortality in older adults with hypertension. Data were obtained from the 2011–2018 National Health and Nutrition Examination Survey (NHANES) and followed for survival through December 31, 2019. DII was calculated by the 24 h dietary history interview. Cox proportional hazards models were used to investigate the associations. A total of 2531 participants were finally included. During a median follow-up of 4.33 years, 471 participants were determined as all-cause mortality. After adjusting for confounding factors, DII was positively correlated with the risk of all-cause mortality (HR = 1.08, 95% CI = 1.01–1.16). Compared with the anti-inflammatory diet group (DII < 0), the pro-inflammatory diet group (DII > 0) had a 54% increased risk of all-cause death (HR = 1.54, 95% CI = 1.13–2.10). The results were robust in subgroup and sensitivity analyses. DII was positively correlated with the all-cause mortality of elderly hypertensive patients. The results provided an aid to dietary evaluation in the nonpharmacologic management of hypertension.

## 1. Introduction

Hypertension is the major cause and preventable risk factor for cardiovascular disease and premature death worldwide [1], and its prevalence increases markedly with advancing age [2]. Data from the Framingham Heart Study showed that the prevalence of hypertension among people aged < 60, 60–79, ≥80 years increased from 27.3% to 63.0% and then from 63.0 to 74.0%, respectively [3]. The current state of the aging population will further exacerbate the problem.

In the past few decades, our understanding of the role of inflammation in health and diet in inflammation has increased rapidly [4]. At the same time, we have increasingly recognized that dietary patterns, i.e., the combined and interacting effects of dietary components, may have a greater influence on disease than individual nutrients. Therefore, the dietary inflammatory index (DII) as a quantitative method to evaluate the level of dietary inflammation emerged as time and conditions required, which could characterize the whole diet of an individual using a quantitative scale from the most anti-inflammatory to the most pro-inflammatory [5,6]. Numerous studies have shown that high DII is associated with the risk or prognosis of most non-communicable diseases, including obesity [7], diabetes [8], cardiovascular disease [9], asthma [10], cancer [11], dementia [12], etc. The DII was also validated to have strong associations with inflammatory biomarkers, including white blood cell counts, c-reactive protein, interleukin-6, and tumor necrosis factor-alpha [13].

The impact of dietary patterns on blood pressure has long been one of the major concerns in public health. Heart-healthy dietary patterns, as an important component of nonpharmacological interventions, have been included in the hypertension management guidelines in recent years [14,15]. The Dietary Approaches to Stop Hypertension (DASH) diet, in conjunction with the reduction of sodium intake, has been shown to be able to lower blood pressure substantially [16]. A Mediterranean diet has also shown favorable effects on blood pressure levels [17]. However, the fixed dietary patterns described above still do not address the problem of a quantitative evaluation of diets. There is a growing body of evidence suggesting that low-grade inflammation plays an important role in initiating and maintaining elevated blood pressure [18]. The DII, which can effectively link diet, inflammation, and health outcomes, has been shown to be associated with the risk of incident hypertension and has the potential to be an effective measure to quantify dietary inflammation levels [19]. However, the role of DII in the prognosis of elderly patients with hypertension remains unclear.

In order to address this knowledge gap, we examined the association between DII, as a quantitative assessment of dietary inflammation levels, and all-cause mortality in a nationally representative sample of older adults (≥65 years old) with hypertension.

## 2. Materials and Methods

### 2.1. Study Design and Participants

The National Health and Nutrition Examination Survey (NHANES) is a cross-sectional survey aiming to investigate the overall health and nutritional status of the non-institutional population in the United States [20]. The protocol of NHANES was approved by the National Centre for Health Statistics (NCHS) ethics review board, and all participants had provided written informed consent.

We identified a total of 39,156 individuals from 4 survey cycles (2011–2018) of NHANES data. The participants younger than 65 years of age (*n* = 33,722) were firstly excluded from our study. The subjects without diagnosed hypertension or survival status (*n* = 13,982) were further excluded. Hypertension was diagnosed by meeting any of the following criteria: systolic blood pressure ≥ 140 and/or diastolic blood pressure ≥ 90 mmHg, self-reported physician diagnosis, and use of antihypertensive medications. Finally, we excluded participants with missing values for DII or any other covariates (*n* = 1319), and a total of 2531 participants were enrolled in our study.

### 2.2. Exposure and Outcome Definitions

All-cause mortality was derived from the records of the National Death Index (NDI) before 31 December 2019, which were linked with NHANES data. Dietary intake was documented utilizing the 24 h dietary history interview and used to calculate DII according to the method published by N. Shivappa et al. [6]. All the dietary data were validated by the Nutrition Methodology Working Group [21]. Twenty-eight food parameters were used to calculate DII in our study, including carbohydrates, protein, total fat, alcohol, fiber, cholesterol, saturated fat, monounsaturated fatty acids, polyunsaturated fatty acids, n-3 fatty acids, n-6 fatty acids, niacin, vitamin A, thiamin (vitamin B1), riboflavin (vitamin B2), vitamin B6, vitamin B12, vitamin C, vitamin D, vitamin E, iron, magnesium, zinc, selenium, folic acid, beta-carotene, caffeine, and energy. The detailed method of the calculation of DII is shown in the Supplementary Method. Previous studies have illustrated the stable predictive ability of DII when using 28 food parameters [22].

### 2.3. Covariates

The demographic information included age, sex, race, marital status, education, smoking, and a ratio of family income to poverty (RIP). The physical examinations and laboratory tests comprised of body mass index (BMI), glycated hemoglobin (HbA1c), alanine aminotransferase (ALT), aspartate aminotransferase (AST), creatinine (Cr), uric acid (UA), triglyceride (TG), total cholesterol (TC), and estimated glomerular filtration rate (eGFR). The dietary composition included sodium and potassium intake. The comorbidities included hyperlipidemia (HL), diabetes (DM), and chronic obstructive pulmonary disease (COPD). The details of relevant definitions are shown in Table S1.

### 2.4. Statistic Analysis

Due to the complex, multistage, stratified, cluster sampling design of the NHANES, proper weighting procedures were employed for all analyses in this study to obtain the whole national estimates [23]. Continuous variables were presented as the weighted mean ± standard error, and categorical variables were expressed as frequency and percentage. A weighted t-test or a chi-square test was used to compare continuous or categorical variables in different groups, respectively.

The association between DII and the risk of all-cause mortality was examined by using a multivariate Cox regression model to estimate hazard ratios (HRs) at a 95% confidence interval (CI). The Schoenfeld residual method was used to examine the proportional hazard assumption. We divided the DII into both pro-inflammatory diets (DII > 0) and anti-inflammatory diets (DII < 0), so that DII could be included into the model in the form of continuous and categorical variables, respectively, to verify the robustness of the results. The baseline variables, with *p* < 0.1, in univariate analysis or related to clinical prognosis would be included in the multivariable COX regression models. We developed three different models to represent hierarchal adjustment for regression models (model 1: unadjusted; model 2: adjusted for sex, age, race, marital status, education, BMI, smoke, and RIP; and model 3: adjusted for sex, age, race, marital status, education, BMI, smoke, RIP, HbA1c, ALT, AST, Cr, TC, sodium, potassium, eGFR, DM, and COPD).

To test the robustness of our findings, stratified analyses were conducted by sex, race, marital status, education, BMI, smoking, diabetes, and COPD. Potential interactions between multiple stratification factors and DII were also examined. We also excluded individuals who died within 2 years of follow-up to partially eliminate the potential reverse causality. The association between DII and all-cause mortality among older adults without hypertension was also examined to provide more indicative information for clinical practice. All analyses in this study were conducted using R (version 4.2.2). Differences were considered statistically significant when the two-sided *p*-value < 0.05.

## 3. Results

### 3.1. Baseline Characteristics of Study Participants

**Figure 1:**
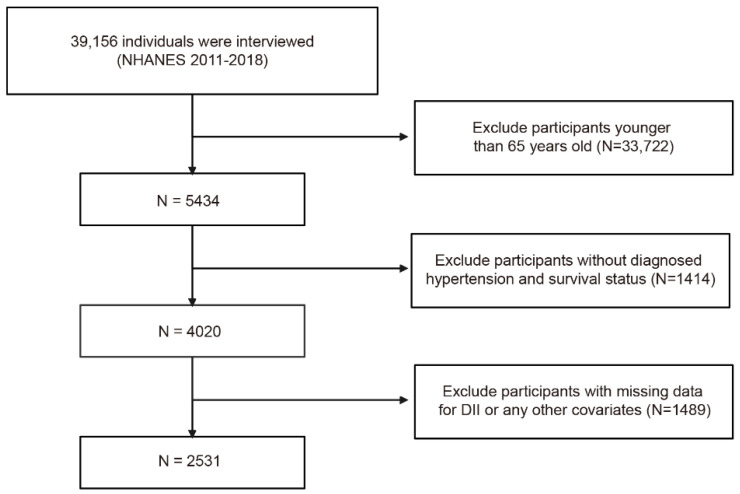
Flow diagram for inclusion of the study population.

**Figure 2:**
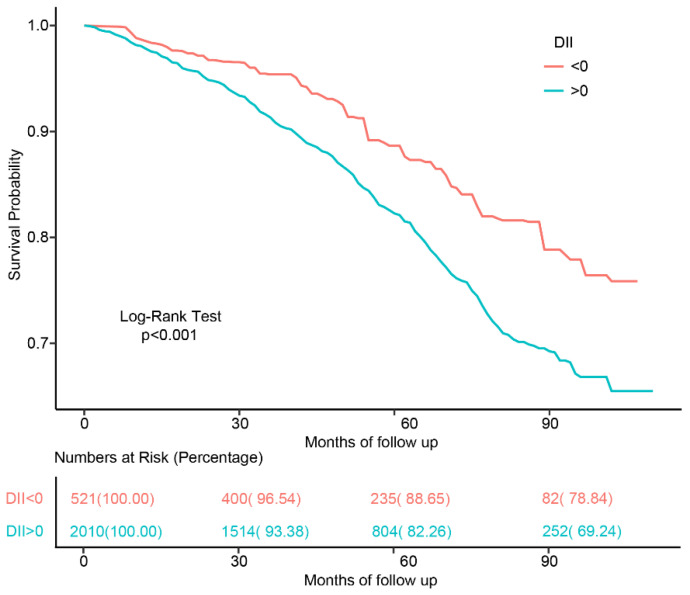
The Kaplan–Meier curve for the study population with different diets.

The process of recruiting is shown in Figure 1. Basic characteristics according to DII and all-cause mortality are summarized, respectively, in Table 1 and Table S2. The present study finally included a total of 2531 participants, of whom 52.27% were female, with an average age of 73.06 ± 0.15 years. After the 52 months of median follow-up duration (interquartile range: 31–76 months), 471 participants were determined as all-cause mortality. The incidence of all-cause death in the pro-inflammatory diet group was 18.03%, significantly higher than the 11.57% in the anti-inflammatory diet group. As shown in Figure 2, the Kaplan–Meier survival curves stratified by DII > and DII < 0 also demonstrated that the pro-inflammatory diet group had higher all-cause mortality (log-rank test, *p* < 0.001). Patients in the pro-inflammatory diet group tended to be female, single, education < high school, black, and had a higher Cr, TC, and a lower RIP (all *p* < 0.05).

### 3.2. Associations between DII and Mortality

As shown in Table 2, DII was significantly associated with an increased risk of all-cause mortality (HR = 1.12, 95% CI = 1.05–1.19) in model 1. The results of the univariate analysis of the COX regression models are shown in Table S3. After multivariable adjustment, the results remained robust and statistically significant with model 2 (HR = 1.11, 95% CI = 1.04–1.18) and model 3 (HR = 1.08, 95% CI = 1.01–1.16). Compared with the anti-inflammatory diet group, all-cause mortality was increased, respectively, by 64, 62, and 54% in the pro-inflammatory diet group in models 1, 2, and 3 (all *p* < 0.05).

### 3.3. Subgroup Analyses and Sensitivity Analyses

**Figure 3:**
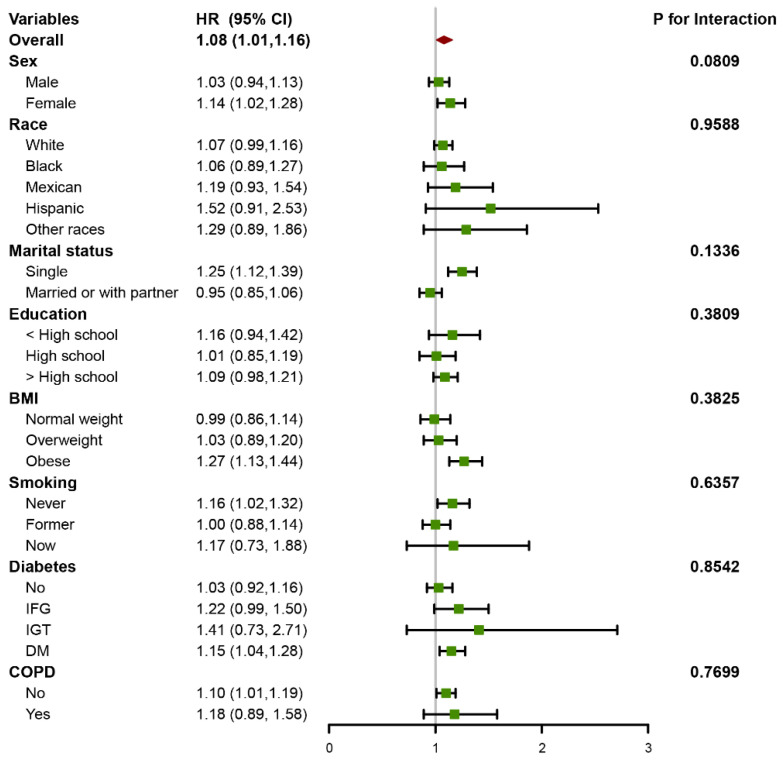
Forest plot for subgroup analysis.

The subgroup analyses in Figure 3 demonstrated that the association of DII and all-cause mortality is consistent and reliable after being stratified by sex, race, marital status, education, BMI, smoking status, diabetes, and COPD (all *p* for interaction > 0.05). It is worth noting that among single and obese individuals, the increase in all-cause mortality risk was particularly significant for each unit increase in the DII. As shown in Table S4, DII remained significantly associated with all-cause mortality after excluding participants who died within 2 years of follow-up. The results shown in Table S5 demonstrated that non-hypertensive older adults with higher DII tended to have higher all-cause mortality, but the association was not statistically significant. It was worth mentioning that the survival probability was significantly reduced in the DII > 0 group after approximately 70 months of follow-up, although the log-rank test showed no difference between the two groups (Figure S1).

Hazard ratios (HRs) were calculated to estimate the association between DII and all-cause mortality using multivariate Cox regression models adjusted for the variables listed in model 3 except for the variable used for stratification.

## 4. Discussion

The present study provided evidence that DII is closely associated with all-cause mortality in a representative sample of U.S. older adults (≥65 years old) with hypertension based on the NHANES database. After adjustment for potential confounders, the pro-inflammatory diet group had a significantly higher risk of all-cause mortality. The finding was consistent and reliable after sensitivity and stratified analyses. As far as we know, this is the first study to explore the relationship between the DII and all-cause mortality among older adults with hypertension.

Chronic low-grade inflammatory responses play an important role in the occurrence and development of hypertension and are closely associated with its deadly complications [18,24]. One of the reasons for this conclusion is that, compared with the general population, hypertensive patients have higher levels of serum inflammatory markers, including C-reactive protein, high-sensitivity C-reactive protein, fibrinogen, and interleukin-6, which are also associated with target organ damage and the risk of future cardiovascular events [25,26]. DII has been proven to be positively correlated with the abovementioned inflammatory markers [13,22,27,28]. Meanwhile, anti-inflammatory dietary patterns, represented by the Mediterranean diet and the DASH diet, can effectively reduce inflammatory marker levels and thus lower blood pressure [29,30,31]. Therefore, diet can participate in the pathogenesis of hypertension as an inflammatory regulator, and its role can be effectively quantified by the DII.

The gut microbiome plays an indispensable mediating role between diet and high blood pressure. Basic research revealed significant differences in gut microbiota between Dahl salt-sensitive and Dahl salt-resistant rats [32]. Meanwhile, the metabolism of commonly used anti-hypertensive drugs was associated with interindividual variation of the gut microbiome [33]. Several microbial metabolites, such as trimethylamine N-oxide and short-chain fatty acids, have been clearly demonstrated to act on downstream cellular targets to participate in the pathogenesis of hypertension [34,35,36]. Dietary contents and quantity play an important role in shaping the composition and function of the human microbiota [37]. Previous research has uncovered that the healthy Mediterranean dietary pattern can exert anti-inflammatory and cardiovascular risk-reducing effects by inducing specific functional and community structures of the gut microbiome [38,39].

Blood pressure management in elderly hypertensive patients has long been considered a challenge since frailty and comorbidities often coexist with hypertension. Frailty can complicate the relationship between blood pressure and mortality, thus making hypertension management more difficult [40]. Research to date has revealed that inflammation is a key contributor to developing frailty [41]. People with high DII were demonstrated more likely to be pre-frail and frail [42]. After adjusting for confounders and frailty, the DII was significantly associated with a long-term mortality risk [43]. Age-related cognitive decline and risk of dementia were considered to be associated with midlife to late-life blood pressure patterns, and the antihypertensive treatment could delay this progression [44,45]. A longitudinal study of 1059 individuals found that diets with high DII scores were associated with an increased risk of incident dementia [12]. Together, the above studies have shown that DII also has an indicative effect on the common complications and comorbidities of hypertension.

In the guidelines for the management of hypertension published in recent years, a healthy diet pattern is one of the main contents of nonpharmacological interventions and has become the first-line therapy for the prevention and treatment of hypertension [14,15]. Therefore, an accurate evaluation of dietary inflammation levels is particularly important. To our knowledge, this was the first large-scale observational study to explore the association between DII and all-cause mortality among elderly hypertensive patients. We conducted analyses using a large, nationally representative sample of the U.S. population and adjusted for demographic, examinational, and laboratory covariates to ensure that the associations are credible and generalizable. Considering the important impact of dietary sodium and potassium intake on blood pressure management [46,47], we included the intake of sodium and potassium from dietary interviews into the model for adjustment. Sensitivity and subgroup analyses further confirmed the robustness of our results.

However, our study had certain limitations. First, although we excluded individuals who died within 2 years of follow-up to reduce reverse causation, the observational study design could not establish genuine causality between DII and all-cause death. Second, the DII calculated by 24 h dietary recall interviews could only represent the habitual diet to a certain extent. The information obtained from the questionnaire inevitably has recall biases, including dietary interviews, smoking, self-reported diagnoses, etc. Third, despite the high completion rate of NHANES, there were still missing values for the analyzed variables, which may reduce the representativeness of the sample.

## 5. Conclusions

In conclusion, we found a positive correlation between the DII and all-cause mortality in hypertensive patients over 65 years old, and the pro-inflammatory diet could increase the mortality risk. These results demonstrated that DII has independent prognostic value and provides assistance in the diet evaluation of elderly hypertensive patients.

## Figures and Tables

**Table 1:** Baseline characteristics of participants.

Variable	Overall	DII < 0	DII > 0	*p*-Value
Age, years	73.06 ± 0.15	72.58 ± 0.30	73.20 ± 0.17	0.071
RIP	2.98 ± 0.07	3.45 ± 0.12	2.84 ± 0.06	<0.001
HbA1c, %	6.04 ± 0.02	5.97 ± 0.05	6.06 ± 0.03	0.132
ALT, IU/L	21.66 ± 0.33	22.71 ± 0.93	21.35 ± 0.35	0.187
AST, IU/L	24.65 ± 0.32	25.15 ± 0.81	24.49 ± 0.38	0.493
Cr, mg/dL	1.03 ± 0.01	0.98 ± 0.02	1.04 ± 0.01	0.004
UA, mg/dL	5.76 ± 0.04	5.73 ± 0.09	5.77 ± 0.04	0.658
TG, mg/dL	157.69 ± 2.74	153.18 ± 5.82	159.04 ± 3.34	0.414
TC, mg/dL	186.24 ± 1.33	178.77 ± 2.71	188.48 ± 1.51	0.003
Sodium, mg/day	3057.03 ± 36.85	3757.25 ± 113.52	2847.15 ± 29.32	<0.001
Potassium, mg/day	2573.34 ± 26.14	3429.53 ± 63.18	2316.71 ± 25.57	<0.001
eGFR, mL/min/1.73 m^2^	68.73 ± 0.42	72.15 ± 0.95	67.71 ± 0.46	<0.001
Sex, *n* (%)				<0.001
Male	1208 (47.73)	314 (56.23)	894 (38.74)	<0.001
Female	1323 (52.27)	207 (43.77)	1116 (61.26)	
Race, *n* (%)				
White	1340 (52.94)	281 (82.64)	1059 (79.66)	0.002
Black	535 (21.14)	89 (5.73)	446 (8.83)	
Mexican	214 (8.46)	48 (3.09)	166 (3.22)	
Hispanic	228 (9.01)	34 (2.08)	194 (3.77)	
Other races	214 (8.46)	69 (6.46)	145 (4.53)	
Marital status, *n* (%)				
Single	1136 (44.88)	180 (29.24)	956 (42.18)	<0.001
Married or with partner	1395 (55.12)	341 (70.76)	1054 (57.82)	
Education, *n* (%)				
<High school	634 (25.05)	104 (11.96)	530 (18.30)	0.002
High school	627 (24.77)	107 (21.71)	520 (26.68)	
>High school	1270 (50.18)	310 (66.33)	960 (55.02)	
BMI, *n* (%)				
Normal weight	565 (22.32)	141 (24.44)	424 (20.15)	0.243
Overweight	924 (36.51)	194 (36.66)	730 (36.94)	
Obese	1042 (41.17)	186 (38.90)	856 (42.92)	
Smoke, *n* (%)				
Never	1240 (48.99)	254 (44.90)	986 (49.95)	0.057
Former	1060 (41.88)	234 (50.09)	826 (42.85)	
Now	231 (9.13)	33 (5.01)	198 (7.20)	
Hyperlipidemia, *n* (%)				
No	376 (14.86)	94 (14.75)	282 (11.67)	0.147
Yes	2155 (85.14)	427 (85.25)	1728 (88.33)	
Diabetes, *n* (%)				
No	1256 (49.62)	277 (54.97)	979 (52.22)	0.261
IFG	162 (6.4)	38 (10.91)	124 (8.25)	
IGT	124 (4.9)	22 (3.48)	102 (4.50)	
DM	989 (39.08)	184 (30.64)	805 (35.03)	
COPD, *n* (%)				
No	2318 (91.58)	484 (92.57)	1834 (90.54)	0.291
Yes	213 (8.42)	37 (7.43)	176 (9.46)	
Status, *n* (%)				
Alive	2060 (81.39)	447 (88.43)	1613 (81.97)	0.002
Death	471 (18.61)	74 (11.57)	397 (18.03)	

Abbreviation: ratio of family income to poverty (RIP), glycated hemoglobin (HbA1c), alanine aminotransferase (ALT), aspartate aminotransferase (AST), creatinine (Cr), uric acid (UA), triglyceride (TG), total cholesterol (TC), estimated glomerular filtration rate (eGFR), body mass index (BMI), impaired fasting glycaemia (IFG), impaired glucose tolerance (IGT), diabetes (DM), and chronic obstructive pulmonary disease (COPD).

**Table 2:** Association between DII and all-cause mortality.

DII	Model 1 HR (95% CI)	Model 2 HR (95% CI)	Model 3 HR (95% CI)
Continuous	1.12 (1.05–1.19)	1.11 (1.04–1.18)	1.08 (1.01–1.16)
DII < 0	Ref = 1.00	Ref = 1.00	Ref = 1.00
DII > 1	1.64 (1.25–2.16)	1.62 (1.21–2.16)	1.54 (1.13–2.10)

Model 1: unadjusted. Model 2: adjusted for sex, age, race, marital status, education, BMI, smoke, and RIP. Model 3: adjusted for sex, age, race, marital status, education, BMI, smoke, RIP, HbA1c, ALT, AST, Cr, TC, sodium, potassium, eGFR, DM, and COPD.

## Data Availability

The datasets analyzed in this study are publicly available and can be found here: https://www.cdc.gov/nchs/nhanes/ (accessed on 22 November 2022).

## Supplementary Materials

The following supporting information can be downloaded at: https://www.mdpi.com/article/10.3390/jcm12020506/s1, Table S1: The detailed definition and classification of covariates; Table S2: Baseline characteristics according to all-cause mortality; Table S3: Univariate analysis of the COX regression model; Table S4: Association between DII and all-cause mortality among patients with hypertension after excluding participants who died within two years of follow-up (*n* = 2399); Table S5: Association between DII and all-cause mortality in the non-hypertensive population; Figure S1: The Kaplan–Meier curve for all-cause mortality in the non-hypertensive population with different diets.
