# Supplementary material for: Dietary Inflammatory Index and All-Cause Mortality in Older Adults with Hypertension: Results from NHANES
Source: J Clin Med. 2023 Jan 7;12(2):506. doi: 10.3390/jcm12020506 (PMC9864621; doi:10.3390/jcm12020506)
Supplement: Supplementary file 1 [file jcm-12-00506-s001.zip › jcm-2081587-supplementary.pdf]

Journal of
Clinical Medicine
MDPI

Supplementary method: The method of the calculation of Dietary Inflammatory Index

The Dietary Inflammatory Index (DII) calculation is standardized to a regionally representative world database which included daily dietary intake from 11 populations all over the world. Both standard mean and standard deviation were provided for all DII food parameters from the world database. For each food parameter, a z-score was created by subtracting the individual's estimated intake from the standard mean. This was then divided by the world standard deviation and then converted to a distribution centered on 0 with bounds between −1 and +1. This value was then multiplied by the inflammatory effect score for each food parameter which was then summed across all food parameters to create the overall DII score.

Table S1. The detail definition and classification of covariates.

Variables	Definitions or Classification
Sex	Male, female.
Race	White, Black, Mexican, Hispanic, Other Races.
Marital status	Single: divorced, separated, widowed and never married;<br>Married or with partner: married and living with partner.
Education	< High school, High school, > High school.
Smoking	Never smoking: <100 cigarettes in lifetime;<br>Former smoking: >100 cigarettes in life and smoke not at all now;<br>Now smoking: >100 cigarettes in lifetime.
RIP	When the ratio of family income to poverty is greater than or equal to 5, the value will be taken as 5.
Weight	Normal: BMI < 25 kg/m²;<br>Overweight: 25 ≤ BMI ≤ 30 kg/m²;<br>Obese: BMI > 30 kg/m².
Laboratory tests	The Specific method can be found in this webpage<br>(https://wwwn.cdc.gov/nchs/nhanes/continuousnhanes/labmethods.aspx?BeginYear=2017)
Sodium	The intake of sodium was determined by means of two days of dietary interviews
Potassium	The intake of potassium was determined by means of two days of dietary interviews
Hyperlipidemia	Hypertriglyceridemia: TG ≥ 150mg/dl;<br>Hypercholesterolemia: TC ≥ 200mg/dl, LDL ≥ 130mg/dl;<br>Low HDL: HDL < 40mg/dl (male),50mg/dl (female);<br>Use of lipid-lowering drugs.
IFG	6.1 mmol/l ≤ Fasting glucose ≤ 7.0 mmol/l.
IGT	7.8 mmol/l ≤ Two-hour OGTT blood glucose ≤ 11.1 mmol/l.
Diabetes	Self-reported doctor diagnosis of diabetes;<br>Glycohemoglobin HbA1c > 6.5%;<br>Fasting glucose ≥7.0 mmol/l;<br>Random blood glucose ≥11.1 mmol/l;<br>Two-hour OGTT blood glucose ≥ 11.1 mmol/l;<br>Use of diabetes medication or insulin.
COPD	FEV1/FVC < 0.7 Post-Bronchodilator;<br>Self-reported doctor diagnosis of emphysema;<br>Subjects who are older than 40 years, have a history of chronic bronchitis, and use the medications including selective phosphodiesterase-4 inhibitors, mast cell stabilizers, leukotriene modifiers and inhaled corticosteroids.

Abbreviation: ratio of family income to poverty (RIP), impaired Fasting Glycaemia (IFG), impaired Glucose Tolerance (IGT), chronic obstructive pulmonary disease (COPD).

Table S2. Baseline characteristics according to all-cause mortality.

Variable	Overall	Alive (2060)	Death (471)	P-value
Age, years	73.06 ± 0.15	72.44 ± 0.16	76.16 ± 0.35	< 0.001
RIP	2.98 ± 0.07	3.07 ± 0.07	2.54 ± 0.09	< 0.001
HbA1c, %	6.04 ± 0.02	6.02 ± 0.02	6.15 ± 0.06	0.039
ALT, IU/L	21.66 ± 0.33	21.92 ± 0.36	20.36 ± 0.70	0.052
AST, IU/L	24.65 ± 0.32	24.37 ± 0.33	26.04 ± 0.67	0.018
Cr, mg/dl	1.03 ± 0.01	0.99 ± 0.01	1.20 ± 0.05	< 0.001
UA, mg/dl	5.76 ± 0.04	5.74 ± 0.04	5.90 ± 0.10	0.138
TG, mg/dl	157.69 ± 2.74	157.86 ± 3.14	156.83 ± 6.55	0.892
TC, mg/dl	186.24 ± 1.33	187.42 ± 1.51	180.33 ± 2.56	0.021
Sodium, mg/day	3057.03 ± 36.85	3084.57 ± 42.89	2918.11 ± 74.40	0.07
Potassium, mg/day	2573.34 ± 26.14	2596.08 ± 29.74	2458.65 ± 52.39	0.029
eGFR, ml/min/1.73 m^2	68.73 ± 0.42	70.33 ± 0.47	60.65 ± 1.10	< 0.001
DII	1.52 ± 0.05	1.46 ± 0.06	1.81 ± 0.10	0.005
Sex, *n* (%)				0.455
Male	1208 (47.73)	957 (42.33)	251 (45.03)	
Female	1323 (52.27)	1103 (57.67)	220 (54.97)	
Race, *n* (%)				0.092
White	1340 (52.94)	1033 (79.58)	307 (84.20)	
Black	535 (21.14)	451 (8.26)	84 (7.39)	
Mexican	214 (8.46)	186 (3.34)	28 (2.43)	
Hispanic	228 (9.01)	202 (3.63)	26 (2.12)	
Other Races	214 (8.46)	188 (5.20)	26 (3.87)	
Marital, *n* (%)				< 0.001
Single	1136 (44.88)	880 (36.94)	256 (50.56)	
Married or with partner	1395 (55.12)	1180 (63.06)	215 (49.44)	
Education, *n* (%)				0.017
< High school	634 (25.05)	490 (15.68)	144 (22.69)	
High school	627 (24.77)	504 (25.25)	123 (26.92)	
> High school	1270 (50.18)	1066 (59.07)	204 (50.39)	
BMI, *n* (%)				< 0.001
Normal weight	565 (22.32)	418 (19.46)	147 (29.58)	
Overweight	924 (36.51)	771 (37.64)	153 (33.02)	
Obese	1042 (41.17)	871 (42.90)	171 (37.40)	
Smoke, *n* (%)				0.068
Never	1240 (48.99)	1035 (49.83)	205 (43.53)	
Former	1060 (41.88)	844 (43.90)	216 (47.67)	
Now	231 (9.13)	181 (6.28)	50 (8.80)	
Hyperlipidemia, *n* (%)				0.277
No	376 (14.86)	302 (12.02)	74 (14.21)	
Yes	2155 (85.14)	1758 (87.98)	397 (85.79)	
Diabetes, *n* (%)				< 0.001
No	1256 (49.62)	1054 (54.80)	202 (43.06)	
IFG	162 (6.4)	138 (9.41)	24 (6.10)	
IGT	124 (4.9)	92 (3.85)	32 (6.35)	
DM	989 (39.08)	776 (31.94)	213 (44.49)	
COPD, *n* (%)				< 0.001
No	2318 (91.58)	1915 (92.72)	403 (82.35)	

Yes	213 (8.42)	145 (7.28)	68 (17.65)

Abbrevation: ratio of family income to poverty (RIP), glycated hemoglobin (HbA1c), alanine aminotransferase (ALT), aspartate aminotransferase (AST), creatinine (Cr), uric acid (UA), triglyceride (TG), total cholesterol (TC), estimated glomerular filtration rate (eGFR), dietary inflammatory index (DII), body mass index (BMI), impaired fasting glycaemia (IFG), impaired glucose tolerance (IGT), diabetes (DM), chronic obstructive pulmonary disease (COPD).

Table S3. Univariate analysis of COX regression model.

Variables	HR (95% CI)	P-Value
Age, years	1.14 (1.11,1.18)	<0.001
RIP	0.84 (0.77,0.91)	<0.001
HbA1c, %	1.17 (1.08,1.27)	<0.001
ALT, IU/L	0.98 (0.96,1.00)	0.039
AST, IU/L	1.01 (1.00,1.02)	0.015
Cr, mg/dl	1.34 (1.23,1.47)	<0.001
UA, mg/dl	1.07 (0.99,1.16)	0.082
TG, mg/dl	1.00 (1.00,1.00)	0.838
TC, mg/dl	1.00 (0.99,1.00)	0.016
Sodium, mg/day	1.00 (1.00,1.00)	0.098
Potassium, mg/day	1.00 (1.00,1.00)	0.014
eGFR, ml/min/1.73 m^2	0.98 (0.97,0.98)	<0.001
DII	1.12 (1.05,1.19)	<0.001
Sex, *n* (%)		
Male	1.00	
Female	0.94 (0.72,1.21)	0.621
Race, *n* (%)		
White	1.00	
Black	0.84 (0.65,1.09)	0.188
Mexican	0.75 (0.49,1.15)	0.193
Hispanic	0.59 (0.39,0.90)	0.014
Other Races	0.75 (0.46,1.21)	0.235
Marital, *n* (%)		
Single	1.00	
Married or with partner	0.59 (0.46,0.76)	<0.001
Education, *n* (%)		
< High school	1.00	
High school	0.89 (0.59,1.34)	0.573
> High school	0.70 (0.51,0.97)	0.030
BMI, n (%)		
Normal weight	1.00	
Overweight	0.65 (0.47,0.89)	0.007
Obese	0.68 (0.50,0.91)	0.009
Smoke, *n* (%)		
Never	1.00	
Former	1.19 (0.94,1.50)	0.146
Now	1.54 (1.02,2.32)	0.039
Hyperlipidemia, *n* (%)		
No	1.00	
Yes	0.89 (0.65,1.21)	0.463
Diabetes, *n* (%)		
No	1	

IFG	1.04 (0.68,1.60)	0.854
IGT	1.41 (0.83,2.41)	0.202
DM	1.80 (1.36,2.38)	<0.001
COPD, *n* (%)		
No	1	
Yes	2.01 (1.47,2.75)	<0.001

Abbrevation: ratio of family income to poverty (RIP), glycated hemoglobin (HbA1c), alanine aminotransferase (ALT), aspartate aminotransferase (AST), creatinine (Cr), uric acid (UA), triglyceride (TG), total cholesterol (TC), estimated glomerular filtration rate (eGFR), dietary inflammatory index (DII), impaired fasting glycaemia (IFG), impaired glucose tolerance (IGT), diabetes (DM), chronic obstructive pulmonary disease (COPD).

Table S4. Association between DII and all-cause mortality among patients with hypertension after excluding participants who died within two years of follow-up (n = 2399).

DII	Model 1 HR (95% CI)	Model 2 HR (95% CI)	Model 3 HR (95% CI)
Continuous	1.11 (1.03,1.20)	1.11 (1.02,1.20)	1.10 (1.01,1.20)
DII < 0	ref = 1.00	ref = 1.00	ref = 1.00
DII > 1	1.66 (1.19,2.30)	1.70 (1.20,2.41)	1.75 (1.25,2.43)

Model 1: unadjusted. Model 2: adjust for sex, age, race, marital status, education, BMI, smoke and RIP. Model 3: adjust for sex, age, race, marital status, education, BMI, smoke, RIP, HbA1c, ALT, AST, Cr, TC, sodium, potassium, eGFR, DM and COPD.

Table S5. Association between DII and all-cause mortality in non-hypertensive population.

DII	Model 1 HR (95% CI)	Model 2 HR (95% CI)	Model 3 HR (95% CI)
Continuous	1.04 (0.91,1.19)	1.05 (0.91,1.21)	1.02 (0.84,1.22)
DII < 0	Ref = 1.00	ref = 1.00	ref = 1.00
DII > 1	1.61 (0.87,2.99)	1.48 (0.82,2.66)	1.56 (0.79,3.08)

The participants younger than 65 years old were firstly excluded(N = 33722). Then the participants diagnosed with hypertension and without survival status were excluded (N = 4036). Last, the participants without missing data (N=869) were included for the analysis. Model 1: unadjusted. Model 2: adjust for sex, age, race, marital status, education, BMI, smoke and RIP. Model 3: adjust for sex, age, race, marital status, education, BMI, smoke, RIP, HbA1c, ALT, AST, Cr, TC, sodium, potassium, eGFR, DM and COPD.

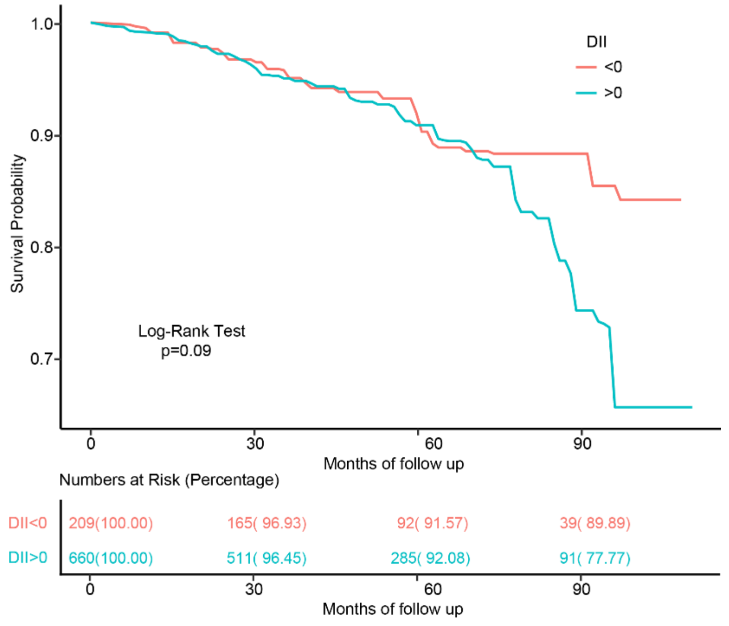

Figure S1. The Kaplan–Meier curve for all-cause mortality in non-hypertensive population with different diets.
